# A rare case of H3K27-altered diffuse midline glioma with multiple osseous and spinal metastases at the time of diagnosis

**DOI:** 10.1186/s12883-023-03135-4

**Published:** 2023-02-28

**Authors:** A. Kaywan Aftahy, Vicki M. Butenschoen, Lisa Hoenikl, Friederike Liesche-Starnecker, Benedikt Wiestler, Friederike Schmidt-Graf, Bernhard Meyer, Jens Gempt

**Affiliations:** 1 Department of Neurosurgery, School of Medicine, Medical Faculty, Klinikum rechts der Isar, Technical University Munich, Ismaninger Str. 22, 81675 Munich, Germany; 2 Department of Neuropathology, School of Medicine, Institute of Pathology, Technical University of Munich, Munich, Germany; 3 Department of Neuroradiology, School of Medicine, Technical University of Munich, Munich, Germany; 4 Neurological Department, School of Medicine, Technical University of Munich, Munich, Germany

**Keywords:** Diffuse midline Glioma, H3K27M, Extraneural metastases, Neuro-oncology

## Abstract

**Background:**

H3K27-altered diffuse midline gliomas are uncommon central nervous system tumors with extremely poor prognoses.

**Case presentation:**

We report the case of a 24-year-old man patient with multiple, inter alia osseous metastases who presented with back pain, hemi-hypoesthesia, and hemi-hyperhidrosis. The patient underwent combined radio-chemotherapy and demonstrated temporary improvement before deteriorating.

**Conclusions:**

H3K27-altered diffuse midline glioma presents an infrequent but crucial differential diagnosis and should be considered in cases with rapid neurological deterioration and multiple intracranial and intramedullary tumor lesions in children and young adults. Combined radio-chemotherapy delayed the neurological deterioration, but unfortunately, progression occurred three months after the diagnosis.

## Background

The majority of spinal cord tumors are low-grade gliomas of astrocytic or ependymal origin, of which 10% are intramedullary astrocytomas. Malignant astrocytomas of the spinal cord have a very poor prognosis [1].

The ratio of low-grade to high-grade astrocytomas in the spinal cord is about 3:1. Glioblastomas represent about 7.5% of all intramedullary gliomas and 1-3% of all spinal cord tumors [2–5].

Extra-cranial metastases of glioblastomas are rare [6–8], approximately 200 published cases emphasize this fact [9, 10]. Several published case reports represent the scarcity of osseous, systemic, or solid metastases [11–14].

H3K27-altered diffuse midline glioma is a newly recognized diffuse high-grade tumor entity in the fifth edition of the WHO Classification of Tumors of the Central Nervous System [15]. It occurs primarily in children. It had been characterized as a diffuse midline glioma with H3K27M mutations in the 2016 WHO classification [1]. Its nomenclature has recently been changed in recognition of other altered mechanisms (e.g., EZHIP protein overexpression) which could underlie the pathogenesis of tumors [16]. Clinically, the diagnostic criteria for H3K27-altered diffuse midline glioma consist of a diffuse growing pattern (i.e., infiltrating), a midline location (including the thalamus, cerebellum, brain stem, and spinal cord), and H3K27-specific neuroglial mutations in addition to supporting histopathological and molecular evidence [15–17].

## Case presentation

### Case history and patient description

A 24-year-old man patient presented with thoracic back pain radiating to his right shoulder. He complained about increased sweating in his right upper body, including his face, as well as the decreased sensation in his right chest. The symptoms progressed over 10 days.

### Physical examination results

The patient described right thoracic pain and gait disturbance, hemi-hypoesthesia, and hemi-hyperhidrosis. Hyperhidrosis can exist on its own or be a symptom of autonomic dysreflexia. Autonomic dysreflexia is a condition that can occur following a T6 or higher-level spinal cord injury. It’s primarily characterized by a sudden spike in blood pressure when areas below the level of injury are stimulated. The pathophysiology may be from local tissue damage causing hyperactivity of preganglionic sympathetic neurons or disinhibition of inhibitory local interneurons [18].

We observed a positive Babinski sign on the right side, increased reflexes of his right arm and leg, and ataxia with the posterior column as the culprit lesion without cranial nerve pathology, motor deficits, or meningism. Bladder dysfunction was intact.

### Imaging

**Fig. 1:**
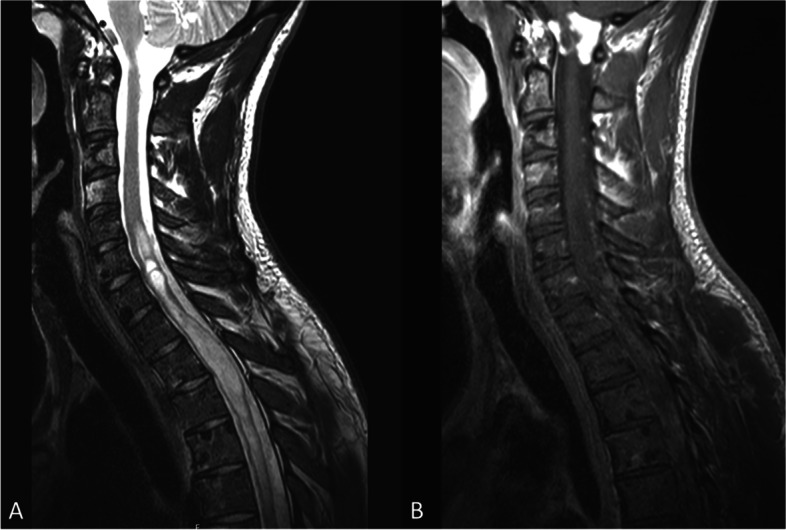
The initial MR spine image presents intra- and extra-medullary metastatic tumor lesions. **A** Sagittal T2-weighted MRI demonstrates diffuse long-distance bulky disease in the spinal cord, as well as diffuse intraosseous lesions. **B** Contrast-enhanced sagittal T1-weighted MRI of the spinal cord shows a contrast-enhanced metastatic lesion at the craniocervical junction

Magnetic resonance imaging (MRI) of the spine and brain was performed and identified a large intramedullary lesion from C5 to Th7 (Fig. 1).

**Fig. 2:**
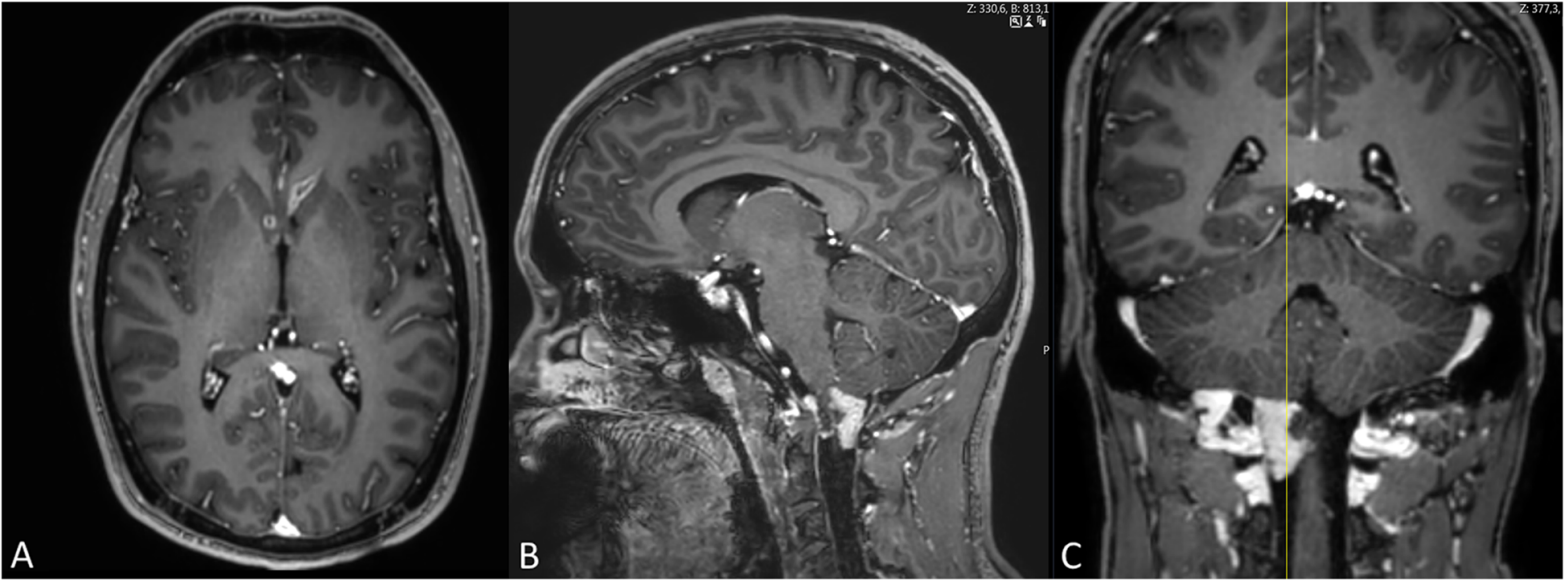
Cerebral contrast-enhanced T1-weighted MR scans present multiple metastatic tumor lesions. **A** contrast-enhanced axial T1-weighted MRI demonstrates lesions at the left lateral ventricle and the right caudate nucleus. **B**, **C** Contrast-enhanced sagittal (**B**) and coronary (**C**) T1-weighted MRI shows a large metastatic lesion at the right craniocervical transition

**Fig. 3:**
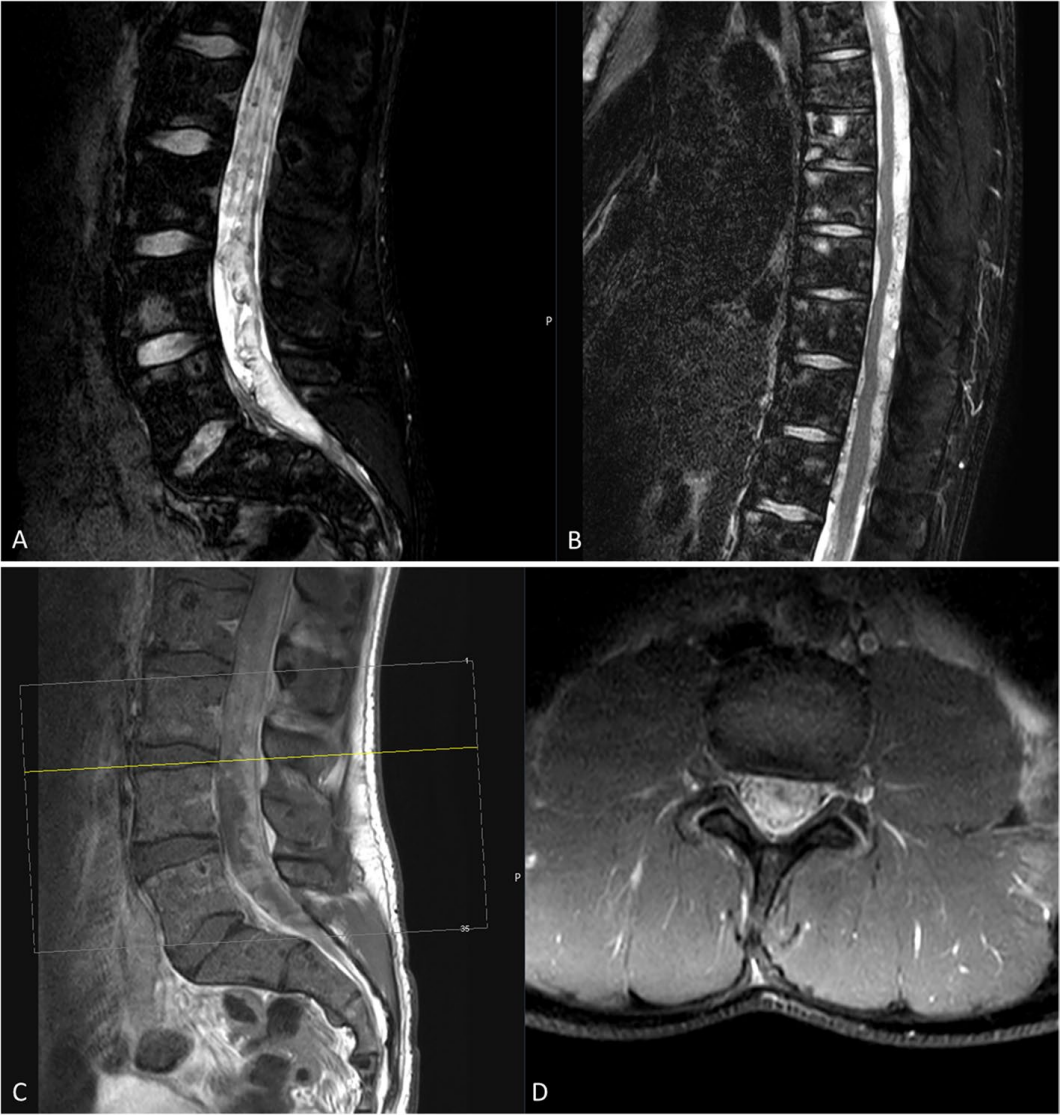
MRI of the thoracic and the lumbar spine. **A**: sagittal T2-weighted MR-image of the lumbar spine demonstrates diffuse metastatic lesions in the conus medullaris and along the cauda equina. **B**: sagittal T2-weighted MRI shows diffuse intradural, extramedullar bulky disease along the thoracic spinal cord. Both images of the thoracic and the lumbar spine present intraosseous tumor lesions. **C**, **D** Contrast-enhanced T1-weighted sagittal (**C**) and axial (**D**) MRI demonstrates diffuse infiltrating bulky tumor-lesions intradural along the spinal cord, the conus medullaris, and the cauda equina

Contrast-enhancing lesions were identified periventricularly (Fig. 2), at the craniocervical junction (Fig. 2), as well as across the spinal cord (Fig. 3). A heterogeneous signal of the vertebrae was suggestive of tumor metastases.

### Computed tomography staging

**Fig. 4:**
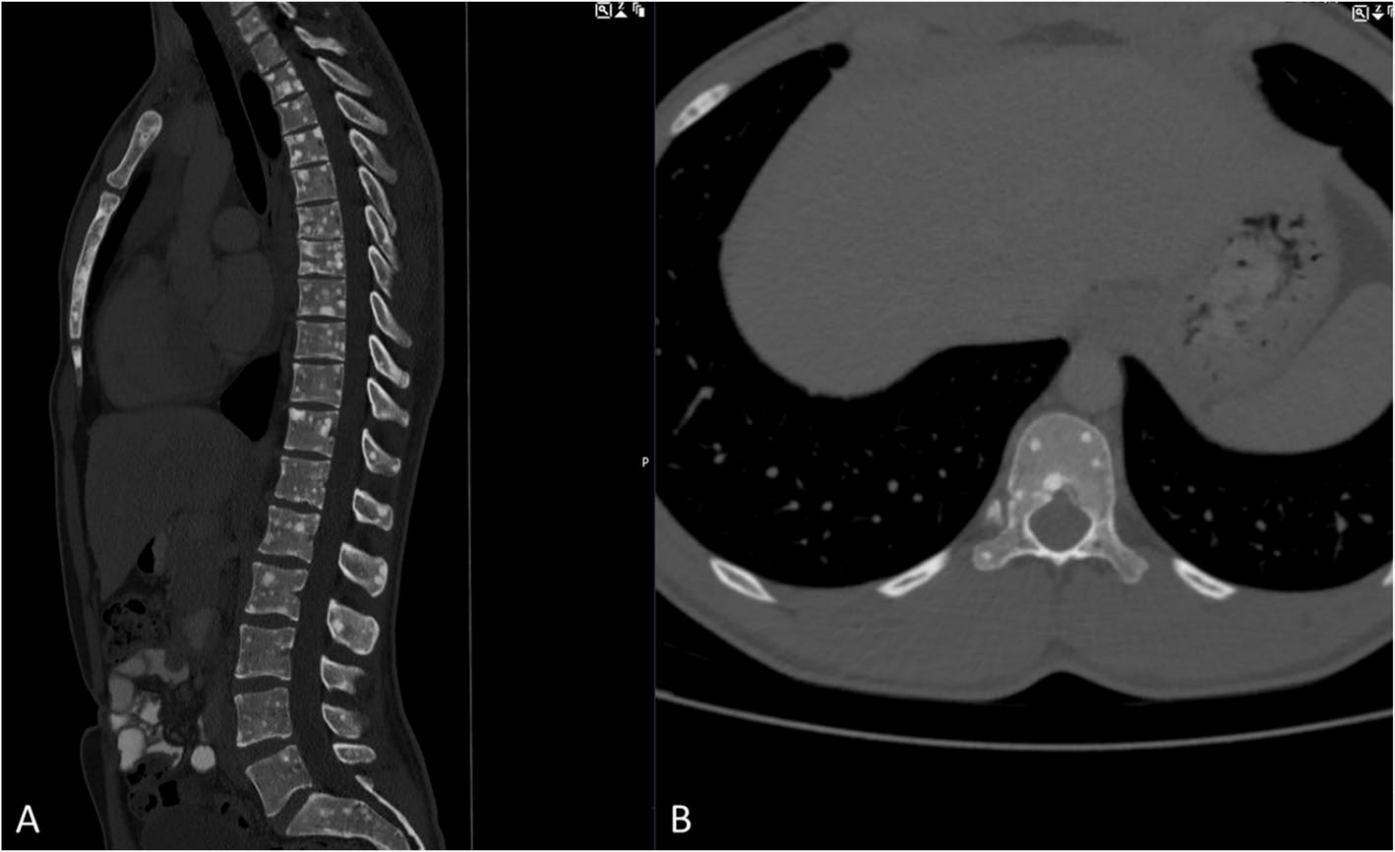
CT images sagittal (**A**) and axial (**B**) of the vertebral column demonstrate diffuse intraosseous lesions along the entire spine (cervical to sacral) and the sternum

Thoracal and abdominal computed tomography staging showed diffuse osseous metastases of the spine and the sternum (Fig. 4). No other tumorous organ lesions were found within the abdominal or chest cavity.

### Cerebrospinal fluid analysis

A lumbar puncture was performed after two days. Cytological analysis of the cerebrospinal fluid (CSF) revealed atypical cells, concerning a leptomeningeal spread. Unfortunately, the number of neoplastic cells was too low, and the fabrication of a cell block for immunohistochemistry could not be performed.

### Biopsy

#### Cranial stereotactic biopsy

**Fig. 5:**
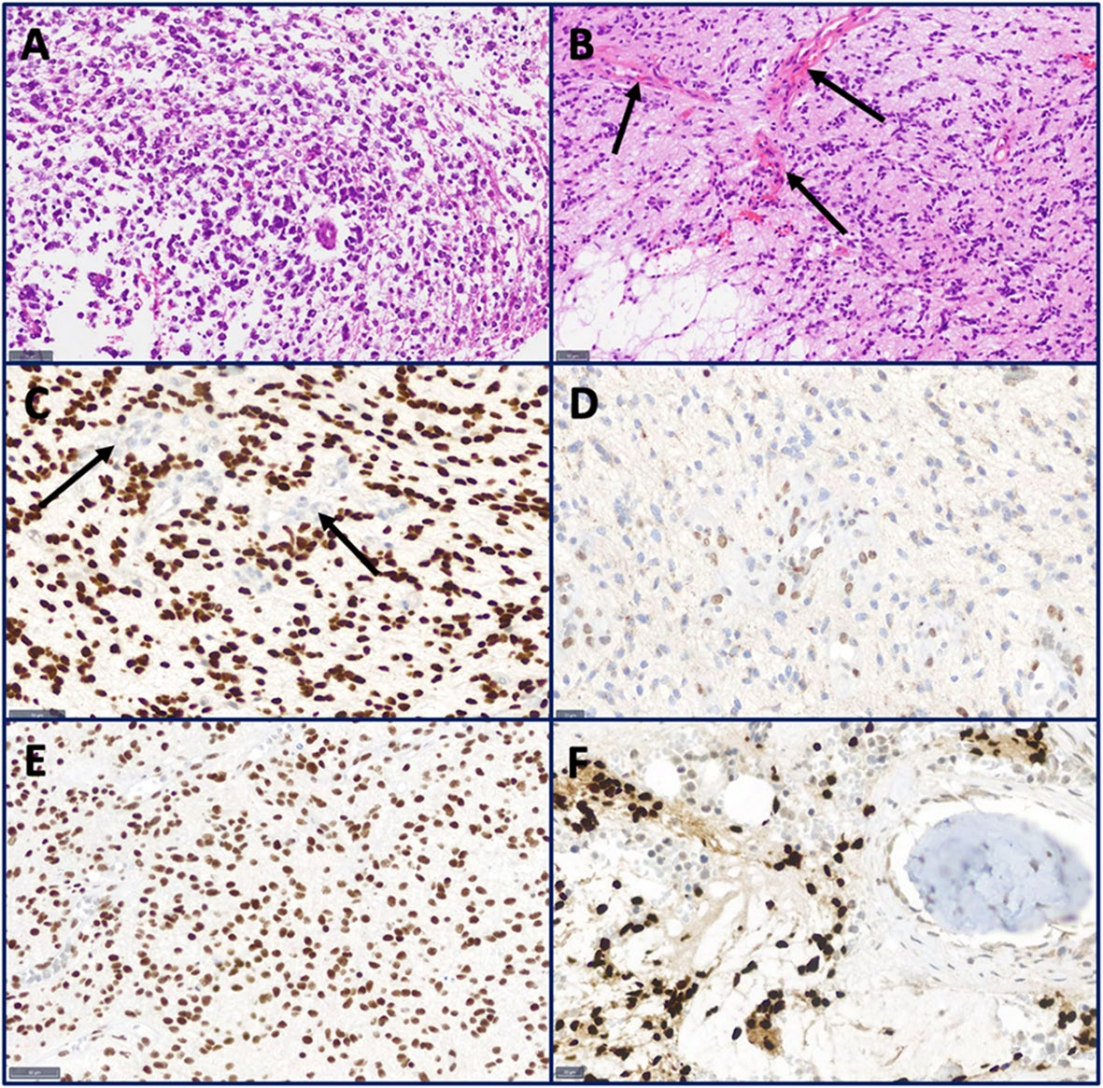
Histological and immunohistochemical analysis. **A** H&E of the cranial stereotactic biopsy shows a tumor with small, round cells. **B** H&E of the lumbar biopsy reveals a partial rhythmic tumor with vascular proliferates (arrows). **C** H3K27M immunohistochemistry shows a strong nuclear positivity of the tumor cells. Endothelial cells of vascular proliferates are negative (arrows). **D** The tumor cells show a loss of the trimethylated lysine (H3K27-m3). **E** The tumor cells are ATRX-positive. **F** H3K27M immunohistochemistry reveals tumor cells in the retrieved bone sample

A stereotactic biopsy of the left frontal contrast-enhancing lesion was performed, revealing a malignant tumor, and predominantly small cells and round cells with mitoses and vascular proliferates were seen (Fig. 5A). Immunohistochemical analyses confirmed a glial origin (detection of MAP 2, Olig2, and GFAP positivity). Molecular analyses could not be conducted due to the small sample size.

#### Lumbar open biopsy

An open biopsy was performed via a hemilaminectomy at L5. The histopathology revealed a glial tumor with a rhythmic growth pattern of moderately pleomorphic tumor cells (Fig. 5B). The mutation of histone 3 (H3K27M, Fig. 5C) with loss of trimethylated lysine (H3K27-m3, Fig. 5D) in that position was detected. ATRX (Fig. 5E) was maintained. H3K27M-mutated tumor cells were detected in the bone sample (Fig. 5F). Molecular analyses detected the isolated loss of chromosome 1 without MGMT promoter methylation. The diagnosis of a H3 K27-altered diffuse midline glioma was made. Supplementary molecular analysis confirmed the results of the immunohistochemistry with the detection of a missense mutation of gene H3F3A (p.K27M and mutation of the FBXW7 gene (p.W486*)).

#### Treatment plan

We opted for combined radiochemotherapy with concomitant temozolomide (75mg/m^2^KOF) following the standard STUPP scheme for malignant brain tumors [19]. The radiation therapy of the complete neuro-axis included the vertebral metastases with a total dose of 36 Gy (fractions of 1.8 Gy). Temozolomide was discontinued after 2 weeks due to a decrease in platelets and white blood cells.

Boosts of intensified radiation were applied at the craniocervical junction and at T1-3 with a total dosage of 45 Gy.

#### The outcome

**Fig. 6:**
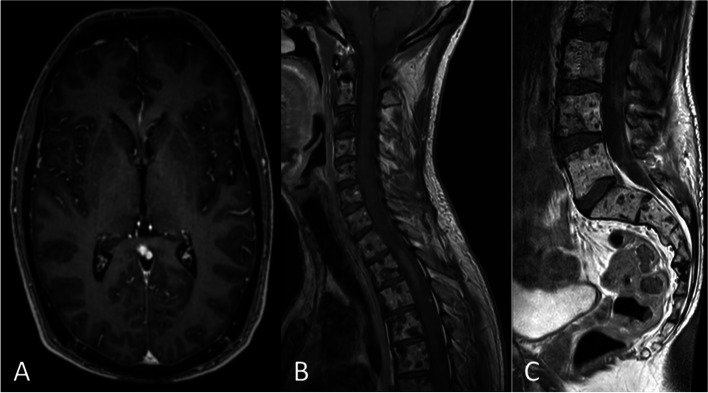
The first follow-up MR after initiation of radio-chemo-therapy shows a partial response of both intracranial (**A**) and intraspinal (**B**, **C**) tumor manifestations. The osseous lesions remained stable

The patient was examined after finishing the radiation therapy. He initially showed improved coordination and gait. A regression of the tumor lesions could be observed on MRI imaging, congruent with the clinical improvement (Fig. 6).

Unfortunately, three months after initial presentation, the patient showed tumor progression with neurological deterioration, and palliative medical care was administered.

## Discussion and conclusions

The combination of the patient’s age, clinical symptoms, and MR imaging first led to the suspected diagnosis of a CNS lymphoma, a germ cell tumor, or a glioma leading to interdisciplinary discussions and, of course, a delay in appropriate treatment.

### Diagnostic pathway

An open lumbar biopsy was performed two weeks later due to the lack of tumor quantity. We later discussed if an initial open biopsy would have shortened the pathway to the final diagnosis.

The prognostic impact of H3K27M mutations remains unclear, as they are uncommon cases of pediatric diffuse astrocytomas with a 10-year survival rate. Unfortunately, in about 50% of cerebrospinal fluid (CSF) samples with leptomeningeal spread, the cell count is not elevated [15].

The anatomical localization complicates bioptic approaches, but Martínez-Ricarte et al. could successfully perform the analysis of IDH1, IDH2, TP53, TERT, ATRX, H3F3A, and HIST1H3B gene mutations through CSF samples. A recent analysis by Panditharatna et al. showed that the mutation was identified in CSF and plasma in 88% of cases. Interestingly, a decrease in H3K27M plasma circulating tumor deoxyribonucleic acid can be a predictor of tumor response to radiotherapy, which was in agreement with 83% of the analyzed patients [20]. Other studies suggest that patients with suspected gliomas at midline structures on MR should first undergo immunohistochemistry testing for H3K27M mutations, especially if tissues were difficult to collect and malignancy is difficult to diagnose based on histological examination alone [16, 21].

### Atypical tumor location

According to cIMPACT-NOW (Consortium to Inform Molecular and Practical Approaches to CNS Tumor Taxonomy – Not Official WHO), the diagnosis of H3 K27-altered diffuse midline glioma includes localization at midline structures [22]. Like heterogeneous histologic features among diffuse midline gliomas, they also have a mixed imaging appearance without distinguishing criteria from histone H3 wild-type diffuse gliomas, [21,46] thus making proper and definite diagnosis aggravating.

### Patient outcome

Usually, rapid neurological deterioration and a fatal outcome within days to months are described [21]. Follow-up imaging initially showed tumor regression on the complete neuro-axis after combined treatment. Unfortunately, tumor progression occurred after 12 weeks, and the patient died 9 months after the presentation in our department. Our patient, therefore, presented an excellent short-term response to therapy, but tumor recurrence and progression occurred after 3 months.

## Conclusions and outlook

A 24-year-old man presented with diffuse neurological symptoms, MR imaging revealed intracerebral, intramedullary, and intraosseous tumor lesions, identified as a diffuse midline glioma with H3K27M mutation. Due to the rarity of this tumor entity and atypical localization, the diagnosis was prolonged. H3 K27-altered diffuse midline glioma presents an infrequent but crucial differential diagnosis and should be considered in cases with rapid neurological deterioration and multiple intracranial and intramedullary tumor lesions in children and young adults. Combined radio-chemotherapy delayed the neurological deterioration, but unfortunately, progression occurred three months after diagnosis.

Based on the location, it is difficult to perform a safe surgical resection or biopsy, which limits routine molecular profiling and treatment options. Noninvasive diagnostic approaches may provide an alternative such as circulating tumor DNA from the biological fluids [23]. Tumor-specific plasma methylomes have been confirmed to distinguish gliomas from extracranial neoplasms. It could be demonstrated that deep sequencing of CSF is a reliable technique for detecting tumor-specific alterations in brain tumors [24]. In 91.9% of cases, at least half of the alterations were identified and the mutation detection by CSF circulating tumor DNA was found to be more sensitive than plasma.

Nevertheless, this diffuse midline glioma is an aggressive disease, and further studies are of utmost importance to better understand its occurrence, and development, and to elaborate more precise treatment strategies.

### Study limitations

Though this was a retrospective case report, we tried to implement a detailed clinical examination and a standardized follow-up protocol based on a certified neuro-oncological board in our clinical workflow. Given the rarity of these lesions prospective inclusion and follow-up is hard to achieve within a reasonable time period. We recommend that multi-center studies should be conducted to address this problem. Another problem in rare entities is reflected by the changing therapy modalities, which may bias the therapy outcome, the learning curve of the treating physicians, multiple physicians involved in the treatment, or changes in the treatment plan.

## Data Availability

The datasets used and/or analysed during the current study are available from the corresponding author on reasonable request.

## Abbreviations

BMP: Bone Morphogenetic Protein
CNS: Central Nervous System
CSF: Cerebrospinal Fluid
CT: Computer Tomography
MRI: Magnet resonance imaging
PNET: Primitive Neuroectodermal Tumor
WHO: World Health Organization
